# Impact of COVID-19 on the Utilization of HIV Testing and Linkage Services in Sierra Leone: Experience from Three Public Health Facilities in Freetown

**DOI:** 10.1007/s10461-023-04149-2

**Published:** 2023-08-29

**Authors:** Sulaiman Lakoh, Moses M. Bangura, Olukemi Adekanmbi, Umu Barrie, Darlinda F. Jiba, Matilda N. Kamara, Daniel Sesay, Abdulai Tejan Jalloh, Gibrilla F. Deen, James B. W. Russell, Ginika Egesimba, George A. Yendewa, Emmanuel Firima

**Affiliations:** 1 College of Medicine and Allied Health Sciences, University of Sierra Leone, Freetown, Sierra Leone; 2 Government of Sierra Leone, Ministry of Health and Sanitation, Freetown, Sierra Leone; 3 Sustainable Health Systems Sierra Leone, Freetown, Sierra Leone; 4 Department of Medicine, College of Medicine, University of Ibadan, Ibadan, Nigeria; 5 Department of Medicine, University College Hospital, Ibadan, Nigeria; 6 Infectious Disease Research Network, Freetown, Sierra Leone; 7 ICAP at Columbia University Mailman School of Public Health, Freetown, Sierra Leone; 8 Department of Medicine, Case Western Reserve University School of Medicine, Cleveland, OH USA; 9 Division of Infectious Diseases and HIV Medicine, University Hospitals Cleveland Medical Center, Cleveland, OH USA; 10 Johns Hopkins Bloomberg School of Public Health, Baltimore, MD USA; 11 Division of Clinical Epidemiology, University Hospital Basel, Basel, Switzerland; 12 Clinical Research Unit, Department of Medicine, Swiss Tropical and Public Health Institute, Basel, Switzerland; 13 University of Basel, Basel, Switzerland; 14 Centre for Multidisciplinary Research and Innovation, Abuja, Nigeria

**Keywords:** COVID-19 pandemic, HIV testing services, HIV testing, Sierra Leone, Linkage to HIV care, Coronavirus 2019

## Abstract

The COVID-19 pandemic adversely affected the delivery of essential health services globally. In this study, we aimed to assess the impact of the pandemic on HIV testing and linkage services at three public health facilities in Freetown, Sierra Leone. We conducted a retrospective study to assess the impact of COVID-19 on HIV testing and linkage to treatment services (HTS) at Connaught Hospital (CH-tertiary), Lumley Government Hospital (LGH-secondary) and George Brooke Community Health Center (GBC-primary) in Freetown. Statistical analyses were conducted in Stata (16.1, StataCorp LLC, College Station, TX). Intra-pandemic HTS (2020) and HTS during recovery (2021) were compared with pre-pandemic HTS (2019). Of the 8538 people tested for HIV in the three facilities, 4929 (57.5%) visited CH. Only 2249 people were tested for HIV in 2020 compared to 3825 in 2019 (difference: − 41.2%, P < 0.001). Fewer people were also tested in 2021 (difference: − 35.6% P < 0.001). The largest reductions in testing in 2020 occurred in women (− 47.7%), children under 15 (− 95.2%), married people (− 42.6%), and CH (− 46.2%). Overall, 1369 (16.0%) people were positive for HIV; CH (878, 17.9%), LGH (469, 15.6%) and GBC (22, 3.5%). The likelihood of a positive HIV test was 26% lower in 2020 than 2019 (PR 0.74; 95% CI 0.64–0.85; P < 0.001), but 16% higher in 2021 than 2019 (PR 1.16; 95% CI 1.03–1.30; P < 0.05). Of the 1369 HIV diagnosis, 526 (38.4%) were linked to care. We found significant disruptions in HIV testing and linkage services at different levels of service delivery during the COVID-19 pandemic, underscoring the need to strengthen essential health services during public health emergencies.

## Background

After it was first reported in China in 2019, and subsequently declared a global pandemic in March 2020 [1, 2], the Coronavirus disease 2019 (COVID-19) prompted countries around the world to institute containment measures such as widespread lockdowns and movement restrictions in an attempt to contain it [3]. As a result, in many countries, access to health care, especially non-COVID-19 care, was compromised, leading to increased morbidity and mortality [4–6].

Following the detection of the first case of COVID-19 in Sierra Leone on March 31, 2020, the government quickly implemented a series of containment measures, including lockdowns, movement restrictions and curfews, similar to the global response to the pandemic [7]. While these restrictive measures were mitigating strategies to contain the spread of the disease and reduce the number of cases requiring treatment at any given time, they also posed considerable challenges to the utilization of essential health services [8].

People living with HIV are a vulnerable population that are likely to be affected by the disruption of services during public health emergencies [9, 10]. Maintaining continuity of HIV care during a pandemic is especially difficult due to limited access to HIV testing and linkage services [10]. HIV testing remains the primary entry point for HIV care and treatment services and its disruption can affect other aspects of the care cascade, including retention and viral suppression [11]. In addition, the emergence of the COVID-19 pandemic has not only increased the difficulties of accessing HIV services, but people living with HIV who often have more comorbidities experience adverse outcomes related to COVID-19 [12]. As a result, the achievement of the new global 95–95–95 target set by UNAIDS was impacted by the COVID-19 pandemic [12].

Despite evidence across the globe indicating a drop in the utilization of testing and other HIV services during the COVID-19 pandemic, low-income countries like Sierra Leone have limited data on the impact of the COVID-19 pandemic on the delivery of healthcare services [13]. Therefore, this study aimed to assess the impact of the COVID-19 pandemic on HIV testing services at three public health facilities at different levels of care (primary, secondary and tertiary care) in Freetown, Sierra Leone.

## Methods

### Design and Setting

**Fig. 1:**
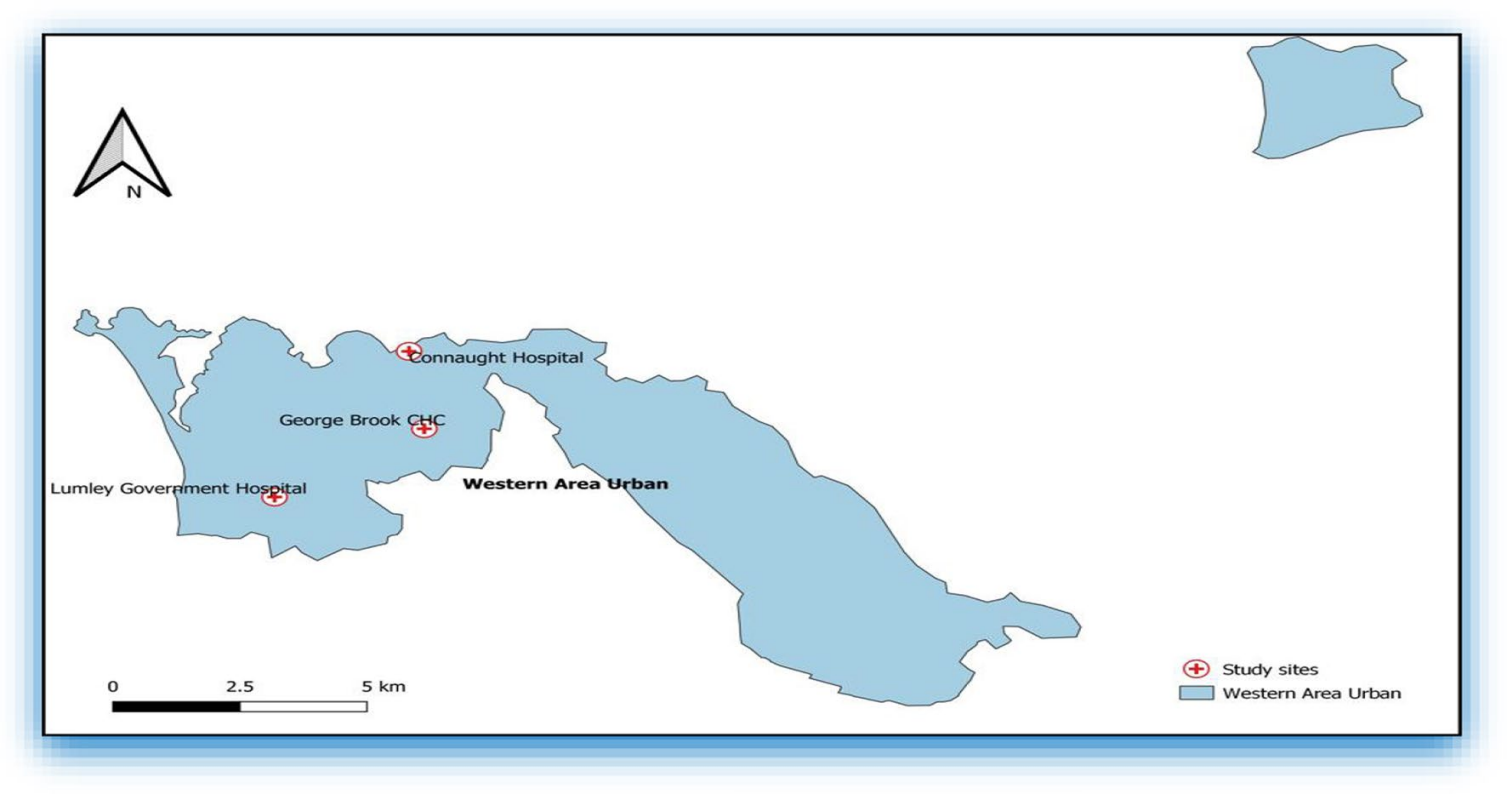
Locations of Connaught Hospital, Lumley Government Hospital and George Brooke Community Health Center

This cross-sectional study was conducted using secondary data of persons tested for HIV at three levels of public health facilities in Freetown, Sierra Leone. The study was conducted among adults and children that were tested for HIV at Connaught Hospital (tertiary facility), Lumley Government Hospital (secondary facility), and George Brooke Community Health Center (primary facility) in Freetown, Sierra Leone’s capital city. While Connaught Hospital (CH) is the main referral hospital in Sierra Leone with a capacity of 300 beds, Lumley Government Hospital (LGH) is a secondary hospital with 32 beds located in the western part of Freetown. George Brooke Community Health Center (CHC) provides primary care services for a large community in Central Freetown. Figure 1 illustrates the locations of the three health facilities in Freetown, where the study was conducted.

### Participant Selection

The dataset for HIV testing and linkage is recorded by the HIV service providers with training on data recording, reporting and quality across all HIV facilities in the country with quality assurance provided by the district and national monitoring and evaluation systems.

We carefully reviewed the HIV testing registers for records of people living with HIV and those who use the facilities solely for the purpose of checking their HIV status between 2019 and 2021. The utilization of services in these hospitals often varies over time periods. To eliminate this seasonal variation in utilization of HIV testing services, we collected data from January through June of each of the three years to enable direct comparison. These periods were well suited to capture the time when the first case of COVID-19 was reported in Sierra Leone. Therefore, we included patients with complete records who had been tested for HIV in three time periods; January to June 2019 (pre-COVID-19 period), January to June 2020 (early intra-COVID-19 period) and January to June 2021 (late intra-COVID-19 period). Records of a few patients with duplicated information, identified through their unique HIV testing codes were excluded. Sociodemographic details (age, sex, and marital status), administrative information (health facility, HIV testing code and entry point) and HIV details (HIV test results, HIV serotype, new HIV diagnosis and linkage to care) were recorded in a Microsoft excel.

### Data Management and Analysis

Data were entered and securely stored in a Microsoft Excel sheet and cross-checked to avoid duplication of variables. Data in Excel format were cleaned, coded and validated prior to analysis.

Descriptive statistics such as mean and standard deviation; and median and interquartile range were used for continuous variables, while frequency and percentage were used for categorical variables. To assess the impact of COVID-19, HIV testing services such as number tested, new diagnoses and new diagnoses linked to care were compared between 2020 and 2019; and between 2021 and 2019. Total percentage differences were then calculated for differences in HIV testing services in the compared years. Percentage differences were also calculated by sex, age categories, marital status, and the three health facilities. Chi-square tests were applied to the differences. Poisson regression was used to determine prevalence ratios for new diagnoses and new diagnoses linked to care between the compared years. P value was set at < 0.05 for all tests. Statistical analyses were conducted in Stata (16.1, StataCorp LLC, College Station, TX).

## Results

A total of 8538 people were tested for HIV in the three facilities over the three time periods. More than half (4929, 57.5%) were tested at Connaught Hospital. In total, there were no disparity in the number of women and men who access HIV testing services. The age distribution and categories were similar across hospitals. The majority (3004, 35%) of people seeking HIV testing were between 25 and 34 (Table 1).

**Table 1:** Characteristics of study participants

Parameter	Total N (%)	CH n (%)	LGH n (%)	Gbc n (%)
Overall total	8572 (100)	4929 (57.5)	3012 (35.1)	631 (7.4)
Sex				
Female	4203 (49.0)	2386 (48.4)	1488 (49.4)	329 (52.1)
Male	4369 (51.0)	2543 (51.6)	1524 (50.6)	302 (47.9)
Age (years)				
< 15	55 (0.6)	27 (0.6)	8 (0.3)	20 (3.2)
15–24	2016 (23.5)	1066 (21.6)	803 (26.7)	147 (23.3)
25–34	3004 (35.0)	1680 (34.1)	1104 (36.6)	220 (34.9)
35–44	1529 (17.8)	907 (18.4)	504 (16.7)	118 (18.7)
≥ 45	1968 (23.0)	1249 (25.3)	593 (19.7)	126 (20.0)
Median (IQR)	32 (25–43)	32 (25–45)	30 (24–40)	30 (24–40)
Marital status				
Single	4555 (53.1)	2642 (53.6)	1619 (53.8)	294 (46.6)
Married	3788 (44.2)	2128 (43.2)	1331 (44.2)	329 (52.1)
Separated/divorced/widowed	211 (2.5)	147 (3.0)	56 (1.9)	8 (1.3)
Missing	18 (0.2)	12 (0.2)	6 (0.2)	0 (0)
Entry				
PICT	8310 (96.9)	4858 (98.6)	2974 (98.7)	478 (75.8)
CICT	107 (1.3)	71 (1.4)	5 (0.2)	31 (4.9)
TB	131 (1.5)	0 (0)	15 (0.5)	116 (18.4)
Others^a^	24 (0.3)	0 (0)	18 (0.6)	6 (0.9)
Tested for HIV				
Yes	8538 (99.6)	4909 (99.6)	2998 (99.5)	631 (100)
No	34 (0.4)	20 (0.4)	14 (0.5)	0
Reasons for testing				
Unprotected sex	6372 (74.3)	2998 (60.8)	2745 (91.1)	629 (99.7)
Optional	2191 (25.6)	1923 (39.0)	266 (8.8)	2 (0.3)
BT/MTC	9 (0.1)	8 (0.2)	1 (0.1)	0 (0)

*CH* Connaught Hospital, *LGH* Lumley Government Hospital, *GBC* George Brooke Community Health Center, *IQR* interquartile range, *PICT* provider initiated counseling and testing, *CICT* client initiated counseling and testing, *TB* tuberculosis, *BT/MTC* blood transfusion/mother-to-child

^a^Others included tests done at immunization points, outpatient departments and walk-in laboratory

**Fig. 2:**
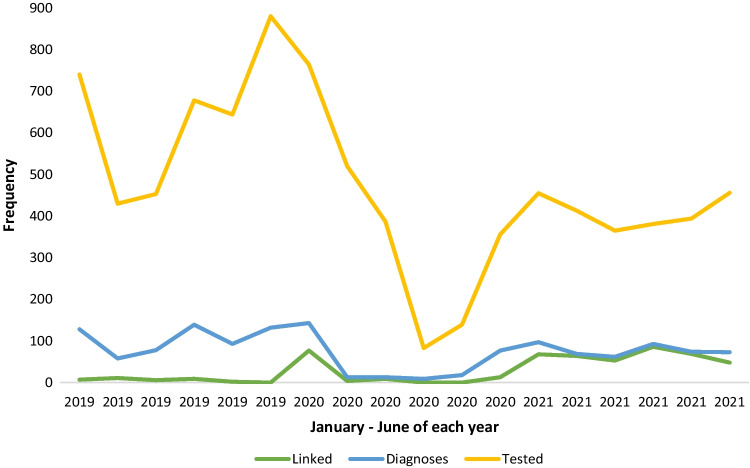
HIV testing services among study participants for first 6 months of 2019, 2020 and 2021

Only 2249 people were tested for HIV in 2020 compared to 3825 in 2019 (percentage difference: − 41.2, P < 0.001). Fewer people were also tested in 2021 (percentage difference: − 35.6, P < 0.001). In 2020, the largest reductions in HIV testing were reported in women (− 47.7%), children under 15 (− 95.2%), married people (− 42.6%), and Connaught Hospital (− 46.2%). A similar reduction in HIV testing services is seen in 2021; however, the percentage is lower compared to 2020 (Table 2). Overall, the trends in HIV testing peaked in June 2019 at 880 tests, before reducing to its lowest level (83 tests) in May 2020 (Fig. 2).

**Table 2:** HIV testing services among study participants in 2020 vs. 2019, and in 2021vs 2019

Parameter	Tested					New HIV diagnosis					Linked to care				
	2019 N	2020 N	2021 N	%change 2020–2019	%change 2021–2019	2019 N (%)	2020 N (%)	2021 N (%)	PR(CI) 2020/2019	PR(CI) 2021/2019	2019 N (%)	2020 N(%)	2021 N (%)	PR(CI) 2020/2019	PR(CI) 2021/2019
Overall total	3825	2249	2464	− 41.2***	− 35.6***	628 (16.4)	273 (12.1)	468 (19.0)	0.74*** (0.64–0.85)	1.16* (1.03–1.30)	35 (5.6)	103 (37.7)	388 (82.9)	6.8*** (4.6–9.9)	14.9*** (10.5–21.0)
Sex															
Female	1932	1011	1244	− 47.7***	− 35.6***	373 (19.3)	160 (15.8)	231 (18.6)	0.82* (0.68.0.99)	0.96 (0.82–1.13)	19 (5.1)	56 (35.0)	189 (81.8)	6.9*** (4.1–11.6)	16.1*** (10.0–25.7)
Male	1893	1238	1219	− 34.6***	− 35.6***	255 (13.5)	113 (9.1)	237 (19.4)	0.68** (0.54–0.85)	1.44*** (1.21–1.72)	16 (6.3)	47 (41.6)	199 (84.0)	6.6*** (3.8–11.7)	13.4*** (8.0–22.3)
Age (years)															
< 15	21	1	31	− 95.2***	+ 47.6***	2 (9.5)	0	2 (6.5)	−	0.68 (0.10–4.81)	0	−	1 (50.0)	−	−
15–24	861	514	636	− 40.3***	− 26.1***	112 (13.0)	60 (11.7)	102 (16.0)	0.90 (0.66–1.23)	1.23 (0.94–1.61)	8 (7.1)	21 (35.0)	83 (81.4)	4.9*** (2.2–11.1)	11.4*** (5.5–23.5)
25–34	1224	845	923	− 31.0***	− 24.6***	216 (17.7)	85 (10.1)	141 (15.3)	0.57*** (0.44–0.73)	0.87 (0.70–1.07)	11 (5.1)	28 (32.9)	122 (86.5)	6.5*** (3.2–12.0)	17.0*** (9.2–31.5)
35–44	733	350	441	− 52.2***	− 39.8***	164 (22.4)	63 (18.0)	132 (29.9)	0.80 (0.60–1.08)	1.34* (1.06–1.68)	9 (5.5)	29 (46.0)	120 (90.9)	8.4*** (4.0–17.7)	16.6*** (8.4–32.6)
≥ 45	986	539	432	− 45.3***	− 56.2***	134 (13.6)	65 (12.1)	91 (21.1)	0.89 (0.66–1.19)	1.55** (1.19–2.02)	7 (5.2)	25 (38.5)	62 (68.1)	7.4*** (3.2–17.0)	13.0*** (6.0–28.5)
Marital status															
Single	1964	1169	1403	− 40.5***	− 28.6***	292 (14.9)	146 (12.5)	210 (15.0)	0.84 (0.69–1.02)	1.00 (0.84–1.20)	14 (4.8)	47 (32.2)	167 (79.5)	6.7*** (3.7–12.2)	16.6*** (9.6–28.6)
Married	1761	1011	1001	− 42.6***	− 43.2***	310 (17.6)	118 (11.7)	241 (24.1)	0.66*** (0.54–0.82)	1.37*** (1.16–1.62)	21 (6.8)	52 (44.1)	205 (85.1)	6.5*** (3.9–10.8)	12.6*** (8.0–19.7)
Separated/divorced/widowed	84	67	60	− 20.2	− 28.6*	25 (29.8)	9 (13.4)	17 (28.3)	0.45* (0.21–0.97)	0.95 (0.51–1.76)	0	4 (44.4)	16 (94.1)	−	−
Hospital															
CH	2385	1284	1240	− 46.2***	− 48.1***	429 (18.0)	176 (13.7)	273 (22.0)	0.76** (0.64–0.91)	1.22** (1.05–1.42)	23 (5.4)	73 (41.5)	230 (84.3)	7.7*** (4.8–12.4)	15.7*** (10.2–24.1)
LGH	1197	803	998	− 32.9***	− 16.6***	199 (16.6)	93 (11.6)	177 (17.7)	0.70** (0.54–0.89)	1.07 (0.87–1.31)	12 (6.0)	26 (28.0)	153 (86.4)	4.6*** (2.3–9.2)	14.3*** (8.0–25.8)
GBC	243	162	226	− 33.3***	− 7.0	0	4 (2.5)	18 (8.0)	−	−	−	4 (100)	5 (27.8)	–	–

*N* number tested, diagnosed or linked to care as appropriate, *PR* prevalence ratio, *CI* confidence interval, *CH* Connaught Hospital, *LGH* Lumley Government Hospital, *GBC* George Brooke Community Health Center

*P < 0.05; **P < 0.01; ***P < 0.001

Across all three facilities, 1369 individuals (16.0%) were HIV positive; CH (878, 17.9%), LGH (469, 15.6%), and GBC (22, 3.5%). Most new HIV diagnoses occurred in 2021 after a dip in 2020. The likelihood of a positive diagnosis was 26% lower in 2020 than 2019 (PR 0.74; 95% CI 0.64–0.85; P < 0.001), but was 16% higher in 2021 than 2019 (PR 1.16; 95% CI 1.03–1.30; P < 0.05). Groups with a lower likelihood of a positive HIV diagnosis in 2020 were men (PR 0.68; 95% CI 0.54–0.85; P < 0.01) and people aged 25–34 (PR 0.57; 95% CI 0.44–0.73; P < 0.001). In 2021 compared to 2019, the HIV positivity was higher in men (PR 1.44; 95% CI 1.21–1.72, P < 0.001), people aged 45 years or older (PR 1.55; 95% CI 1.19–2.02; P < 0.01), married people (PR 1.37; 95% CI 1.16–1.62; P < 0.001), and CH (PR 1.22; 95% CI 1.05–1.42; P < 0.01).

Of the 1369 HIV positive patients, 526 (38.4%) were linked to care; CH (326, 37.1%), LGH (191, 40.7%) and GBC (9, 40.9%). Overall, and consistently within the groups, linkage to care was higher in 2020 and 2021, compared to 2019 (Table 2). In April 2020, new HIV cases reduced to nine, and there were no newly diagnosed HIV patients linked to care in April 2020 and May 2020 (Fig. 1).

## Discussion

This study reports on the impact of the COVID-19 pandemic on HIV testing and linkage to HIV treatment services provided at different levels of health care in Sierra Leone. We observed an overall decline of HIV testing services of 41.2% between 2019 (pre-COVID-19) and 2020 (early intra-COVID-19) and 35.7% between 2019 and 2021 (late intra-COVID-19). The Global Fund for HIV, TB, and Malaria's 2021 report on the impact of COVID-19 on the continuity of health services in 32 low- and middle-income countries shows that the continuity of HIV services was severely disrupted by the COVID-19 pandemic as they observed similar reductions of 41% in the utilization of HIV testing services between 2019 and 2020 [14]. The COVID-19 pandemic has led to different adaptations and changes in health-seeking behavior among populations around the world, resulting in a significant decline in HIV service utilization [15, 16]. Thus, regardless of the setting in low- and middle-income countries, this finding shows that public health emergencies can have a greater impact on the essential health services and calls for a coordinated approach to build resilient health systems to withstand health security threats and mitigate the impact of future public health events on the utilization of essential health services in these countries.

A study in the United States of America (USA) found a 56% decline in HIV testing services between 2019 and 2020, higher than the 41.2% decline reported in our study [17]. Similarly, in China, HIV testing services fell by 49% in the first three months of the introduction of COVID-19 prevention measures [18]. This variation could be explained from the fact that, in Africa, due to the African paradox, there were relatively fewer COVID-19 cases, and a lower-case fatality rate compared with China and USA, resulting in less severe restrictions and less fear among the populace to access HIV testing services [19–22]. Differences in COVID-19-related death rates across countries have greater implications for global policy. Most cases of COVID-19 in African countries such as Sierra Leone were managed in the community or home settings as they were mild or asymptomatic, allowing health facilities to focus on the provision of health services such as HIV testing services.

The overall HIV prevalence of 16% reported in this study was higher than the 6% reported in ten secondary and primary care settings in Sierra Leone, but lower than the level previously reported by the national referral hospital (24.3%) [23, 24]. Nonetheless, an HIV prevalence of 16% reported in this study was higher than the national HIV prevalence of 1.7% reported in 2019 [25]. This can be explained by the concentration of HIV cases, as most people tested at healthcare facilities were seriously ill. Overall, the higher prevalence of HIV in the tertiary hospital reflects on the challenges of HIV service delivery in this setting, including low viral suppression and high prevalence of late-stage or advanced HIV disease, and opportunistic fungal and non-fungal infections [26–30].

We observed that the likelihood of a positive HIV test was 16% higher during the COVID-19 pandemic in 2021 than before the COVID-19 pandemic in 2019, in contrast to the non-significant increase in HIV positivity reported by Moitra and colleagues [31]. The reason for the increased HIV positivity rate is unclear, but as stated by Moitra et al., it could be that persons seeking testing during the COVID-19 pandemic may have been symptomatic or assessed themselves to be at high risk for HIV infection [31] or perhaps may reflect an increase in the prevalence of HIV in the general population of Sierra Leone. Therefore, the national HIV program should strengthen public education and strategies for targeted HIV testing and linkage services in the wake of COVID-19 pandemic and other public health emergencies.

Children and women are particularly vulnerable to the impact of COVID-19 on HIV testing services. Barriers such as fear of exposure to COVID-19, limited access to services, and disruption of social life and livelihoods are among the factors affecting service utilization by women and children [32, 33]. In this study, HIV testing services for children under 15 years and female population declined by 95% and 45% between 2019 and 2020, respectively. These findings underscore the need to pay special attention to vulnerable populations like women and children during public health emergencies.

Our study shows that HIV testing services were significantly affected at all levels of care, from tertiary to primary care. Before Sierra Leone reported its first case of COVID-19 in March 31, 2020, hospital adaptations, such as a planned reconfiguration of health services, led to significant reductions in hospital HIV services [34]. This is reflected in a larger decline in HIV testing in the tertiary hospital (− 48.1%) than in primary health settings (− 7%) between 2020 and 2021.

In 2019, the National HIV Program added information about linkage of people who test positive for HIV to the HIV Testing Register. The introduction of this information is expected to increase the linkage of HIV-positive people to treatment, care and support services. In the present study, there was a decline in the level of linkage to HIV treatment, care and support services, during the COVID-19 period similar to reports in China [18]. This reflects on the low utilization of HIV testing services. Linkage to HIV testing services is crucial for the HIV care continuum and achievement of the ambitious global target of 95–95–95 by 2030.

Our study had limitations, including those inherent in retrospective studies such as missing variables in a small number of patients. Second, our study was conducted in healthcare facilities in Freetown, Sierra Leone’s capital. COVID-19 restrictions vary, with urban settings such as Freetown having stricter restrictions than rural settings. Therefore, the impact of COVID-19 on HIV testing services in rural areas may be lower than that reported in our study, making it possible that our findings cannot be easily generalized to wider Sierra Leone. Analysis of program data will provide more information on the extent of the impact of COVID-19 on HIV testing services across the country. Despite these limitations, the information provided by our study will inform policy development and implementation of the HIV response in Sierra Leone.

## Conclusion

We found significant disruptions in HIV testing services at different levels of service delivery during the COVID-19 pandemic, underscoring the need to strengthen essential health services on the global and national agendas of the public health emergency. Furthermore, there is a need to strengthen differentiated testing services for HIV diagnoses in vulnerable populations such as children and women.

## Data Availability

The data supporting this article is available in the repository of University of Sierra Leone and will be made available on request to the corresponding authors when required.

## Abbreviations

BMI: Body mass index
HIV: Human immunodeficiency virus
MTB: *Mycobacterium tuberculosis*
TB: Tuberculosis
WHO: World Health Organization

## References

[1] World Health Organization. Coronavirus disease (COVID-2019) Situation Reports. https://www.who.int/europe/emergencies/situations/covid-19. Accessed 16 Oct 2020.

[2] Huang C, Wang Y, Li X, Ren L, Zhao J, Hu Y, Zhang L, Fan G, Xu J, Gu X, Cheng Z, Yu T, Xia J, Wei Y, Wu W, Xie X, Yin W, Li H, Liu M, Xiao Y, Gao H, Guo L, Xie J, Wang G, Jiang R, Gao Z, Jin Q, Wang J, Cao B. Clinical features of patients infected with 2019 novel coronavirus in Wuhan, China. Lancet. 2020;395(10223):497–506. 10.1016/S0140-6736(20)30183-5. Erratum in: Lancet. 2020. 10.1016/S0140-6736(20)30183-5 PMC7159299 31986264

[3] Joffe AR (2021). COVID-19: Rethinking the lockdown groupthink. Front Public Health..

[4] Kidd M (2020). Australia’s primary care COVID-19 response. Aust J Gen Pract.

[5] Birkmeyer JD, Barnato A, Birkmeyer N, Bessler R, Skinner J (2020). The impact of the COVID-19 pandemic on hospital admissions in the United States. Health Aff (Millwood).

[6] Abou Ghayda R, Lee KH, Han YJ, Ryu S, Hong SH, Yoon S, Jeong GH, Yang JW, Lee HJ, Lee J, Lee JY, Effenberger M, Eisenhut M, Kronbichler A, Solmi M, Li H, Jacob L (2022). The global case fatality rate of coronavirus disease 2019 by continents and national income: a meta-analysis. J Med Virol.

[7] Lakoh S, Jiba DF, Baldeh M, Adekanmbi O, Barrie U, Seisay AL, Deen GF, Salata RA, Yendewa GA (2021). Impact of COVID-19 on tuberculosis case detection and treatment outcomes in Sierra Leone. Trop Med Infect Dis.

[8] MacIntyre CR, Heslop DJ (2020). Public health, health systems and palliation planning for COVID-19 on an exponential timeline. Med J Aust.

[9] Jiang H, Zhou Y, Tang W (2020). Maintaining HIV care during the COVID-19 pandemic. Lancet HIV.

[10] The Lancet HIV. When pandemics collide. Lancet HIV. 2020;7(5):e301. 10.1016/S2352-3018(20)30113-2 10.1016/S2352-3018(20)30113-2 PMC7194968 32339471

[11] Ambrosioni J, Blanco JL, Reyes-Urueña JM, Davies MA, Sued O, Marcos MA, Martínez E, Bertagnolio S, Alcamí J, Miro JM, COVID-19 in HIV Investigators (2021). Overview of SARS-CoV-2 infection in adults living with HIV. Lancet HIV..

[12] Celum C, Barnabas R (2019). Reaching the 90–90–90 target: lessons from HIV self-testing. Lancet HIV.

[13] Moynihan R, Sanders S, Michaleff ZA, Scott AM, Clark J, To EJ, Jones M, Kitchener E, Fox M, Johansson M, Lang E, Duggan A, Scott I, Albarqouni L (2021). Impact of COVID-19 pandemic on utilisation of healthcare services: a systematic review. BMJ Open..

[14] Impact of COVID-19 on HIV, TB and Malaria and systems for health—a Global Fund for HIV, TB and Malaria snapshot report. https://www.theglobalfund.org/media/10776/covid-19_2020-disruption-impact_report_en.pdf. Accessed 23 Feb 2023.

[15] Chanda-Kapata P, Ntoumi F, Kapata N, Lungu P, Mucheleng'anga LA, Chakaya J, Tembo J, Himwaze C, Ansumana R, Asogun D, Mfinanga S, Nyasulu P, Mwaba P, Yeboah-Manu D, Zumla A, Nachega JB (2022). Tuberculosis, HIV/AIDS and malaria health services in sub-Saharan Africa—a situation analysis of the disruptions and impact of the COVID-19 pandemic. Int J Infect Dis.

[16] Mhango M, Chitungo I, Dzinamarira T (2020). COVID-19 lockdowns: impact on facility-based HIV testing and the case for the scaling up of home-based testing services in sub-Saharan Africa. AIDS Behav.

[17] O'Grady TJ, Yuan Y, Harris JM, Massaroni RJ, Fuller JA, Tesoriero JM (2023). Impact of COVID-19 on HIV testing among AIDS Institute-funded providers in New York state—a time series analysis. J Acquir Immune Defic Syndr.

[18] Shi L, Tang W, Hu H, Qiu T, Marley G, Liu X, Chen Y, Chen Y, Fu G (2021). The impact of COVID-19 pandemic on HIV care continuum in Jiangsu, China. BMC Infect Dis.

[19] World Health Organization. WHO Coronavirus Disease (COVID-19) Dashboard; 2020. https://covid19.who.int/. Accessed 10 June 2020.

[20] Thornton J (2020). COVID-19: Africa’s case numbers are rising rapidly, WHO warns. BMJ..

[21] Lawal Y (2021). Africa’s low COVID-19 mortality rate: a paradox?. Int J Infect Dis.

[22] Abou Ghayda R, Lee KH, Han YJ, Ryu S, Hong SH, Yoon S, Jeong GH, Yang JW, Lee HJ, Lee J, Lee JY, Effenberger M, Eisenhut M, Kronbichler A (2022). The global case fatality rate of coronavirus disease 2019 by continents and national income: a meta-analysis. J Med Virol.

[23] Lakoh S, Firima E, Jiba DF, Sesay M, Conteh MM, Deen GF (2019). Low partner testing in high HIV prevalence setting in Freetown, Sierra Leone: a retrospective study. BMC Res Notes.

[24] Kassa G, Dougherty G, Madevu-Matson C, Egesimba G, Sartie K, Akinjeji A, Tamba F, Gleason B, Toure M, Rabkin M (2020). Improving inpatient provider-initiated HIV testing and counseling in Sierra Leone. PLoS ONE.

[25] Sierra Leone Demographic Health survey 2019. https://dhsprogram.com/pubs/pdf/FR365/FR365.pdf. Accessed 24 Mar 2023.

[26] Yendewa GA, Poveda E, Lakoh S, Yendewa SA, Jiba DF, Salgado-Barreira A, Sahr F, Salata RA (2018). High prevalence of late-stage disease in newly diagnosed human immunodeficiency virus patients in Sierra Leone. Open Forum Infect Dis.

[27] Lakoh S, Jiba DF, Kanu JE, Poveda E, Salgado-Barreira A, Sahr F, Sesay M, Deen GF, Sesay T, Gashau W, Salata RA, Yendewa GA (2019). Causes of hospitalization and predictors of HIV-associated mortality at the main referral hospital in Sierra Leone: a prospective study. BMC Public Health.

[28] Lakoh S, Orefuwa E, Kamara MN, Jiba DF, Kamara JB, Kpaka S, Denning DW (2021). The burden of serious fungal infections in Sierra Leone: a national estimate. Ther Adv Infect Dis.

[29] Lakoh S, Rickman H, Sesay M, Kenneh S, Burke R, Baldeh M, Jiba DF, Tejan YS, Boyle S, Koroma C, Deen GF, Beynon F (2020). Prevalence and mortality of cryptococcal disease in adults with advanced HIV in an urban tertiary hospital in Sierra Leone: a prospective study. BMC Infect Dis.

[30] Lakoh S, Jiba DF, Adekanmbi O, Poveda E, Sahr F, Deen GF, Foray LM, Gashau W, Hoffmann CJ, Salata RA, Yendewa GA (2020). Diagnosis and treatment outcomes of adult tuberculosis in an urban setting with high HIV prevalence in Sierra Leone: a retrospective study. Int J Infect Dis.

[31] Moitra E, Tao J, Olsen J (2022). Impact of the COVID-19 pandemic on HIV testing rates across four geographically diverse urban centres in the United States: an observational study. Lancet Reg Health Am.

[32] Posada R, Waldman R, Chory A, Martin R, Cohen A, Chiacchia S, Childs J, Enane LA, Vreeman R (2022). Longitudinal impacts of the COVID-19 pandemic on adolescents living with HIV in New York City. AIDS Care.

[33] Kumar N, Mangla M (2022). Influence of paired pandemic of COVID-19 and HIV infection on pregnant women and children: a challenging issue. J Mother Child.

[34] Sevalie S, Youkee D, van Duinen AJ, Bailey E, Bangura T, Mangipudi S, Mansaray E, Odland ML, Parmar D, Samura S, van Delft D, Wurie H, Davies JI, Bolkan HA, Leather AJM (2021). The impact of the COVID-19 pandemic on hospital utilisation in Sierra Leone. BMJ Glob Health.

